# Role of the *nagZ* Gene in Antibiotic Resistance and Environmental Stress Tolerance of *Yersinia enterocolitica*

**DOI:** 10.3390/microorganisms14102398

**Published:** 2026-10-20

**Authors:** Qianwen Pan, Yumeng Dong, Qixin Chen, Ruohan Li, Zhuo Yue, Xiaoya Tian, Jingyu Chen

**Affiliations:** 1 Key Laboratory of Food Bioengineering (China National Light Industry), College of Food Science and Nutritional Engineering, China Agricultural University, Beijing 100083, China; 2 Sichuan Advanced Agricultural & Industrial Institute, China Agricultural University, Chengdu 611430, China

**Keywords:** *nagZ*, *Yersinia enterocolitica*, N-acetylglucosamine, antibiotic resistance, environmental stress tolerance

## Abstract

NagZ is a glycoside hydrolase that regulates AmpC β-lactamase and biofilm formation in Gram-negative bacteria, and its inhibitors show promise as antibiotic adjuvants. However, the role of *nagZ* in the important foodborne pathogen *Yersinia enterocolitica*, which exhibits strong environmental adaptability and increasing rates of antibiotic resistance, remains unclear. This study investigated the impact of NagZ on antibiotic resistance and environmental tolerance in *Y. enterocolitica*. The minimum inhibitory concentrations (MICs) of relevant strains against ampicillin, ceftazidime, and fosfomycin sodium were determined. Since NagZ hydrolyzes peptidoglycan fragments bearing N-acetylglucosamine (GlcNAc), the synergistic effects of exogenous GlcNAc on resistance phenotypes were further evaluated. Environmental stress assays were also performed to evaluate the contribution of NagZ to environmental tolerance. The results showed that deletion of *nagZ* produced a pronounced increase in susceptibility to ampicillin, whereas MIC shifts for ceftazidime and fosfomycin sodium were within the experimental error. For ampicillin, exogenous GlcNAc reduced the MIC from 64 µg/mL (WT) to 16 µg/mL and further to 2 µg/mL in the *nagZ* mutant background. The Δ*nagZ* mutant also displayed impaired biofilm formation with 23.91%, 25.42%, and 16.46% reductions relative to WT at 24, 48, and 72 h. Compared with WT, the Δ*nagZ* mutant exhibited relative survival reductions of 32.59% (42 °C), 31.91% (4 °C), 36.37% (SDS), and 71.27% (oxidative stress). These findings indicate that NagZ may represent a critical node in the regulatory network linking antibiotic resistance and environmental adaptation, highlighting its potential as a novel target for controlling this foodborne pathogen.

## 1. Introduction

Foodborne pathogens with escalating antimicrobial resistance pose a major challenge to food safety and a significant threat to public health. *Yersinia enterocolitica*, a typical enteropathogenic foodborne zoonotic pathogen, is widely distributed in nature and can infect humans via the fecal–oral route through contaminated food and water, causing diarrhea and even systemic infections [1,2]. *Y. enterocolitica* exhibits remarkable environmental adaptability. It is capable of slow growth at 0–4 °C, with a reported minimum growth temperature near 0 °C [3]. *Y. enterocolitica* is heat-sensitive and readily inactivated by pasteurization; thermal inactivation kinetics show D-values of 1.6 min at 58 °C, 0.45 min at 60 °C, and 0.18 min at 62 °C [4]. Nevertheless, its cold-adapted physiology enables persistence and slow growth during cold storage, complicating its control during cold-chain management. Concurrently, the species exhibits intrinsic resistance to penicillins and first-generation cephalosporins, including ampicillin and cephalothin, which is mediated by two chromosomally encoded β-lactamases, BlaA and BlaB [5,6,7,8]. The combination of cold tolerance, strong environmental adaptability, and both intrinsic and acquired resistance mechanisms significantly elevates the safety risks associated with this pathogen, substantially undermining the efficacy of traditional physical and chemical control measures and complicating clinical treatment [9]. Therefore, identifying effective antimicrobial targets against this pathogen has become a research priority in both the food industry and the field of public health prevention.

The β-N-acetylglucosaminidase encoded by the *nagZ* gene is a cytoplasmic glycoside hydrolase that plays a central role in the peptidoglycan recycling pathway of Gram-negative bacteria. It removes GlcNAc from GlcNAc-1,6-anhydro-MurNAc-peptides by hydrolyzing the β-1,4-glycosidic bond, releasing GlcNAc and 1,6-anhydro-MurNAc-peptides(anhMurNAc-peptides) [10]. In recent years, the functional characterization of NagZ has expanded beyond its initial role in antibiotic resistance regulation to encompass virulence and biofilm modulation. In *Pseudomonas aeruginosa*, NagZ inactivation prevents and reverses β-lactam resistance [11]; in *Enterobacter cloacae*, NagZ inactivation significantly attenuates virulence in multiple infection models [12]; and in *Neisseria gonorrhoeae*, NagZ exhibits activity in disassembling pre-formed biofilms [13]. Although recent studies have reported that cinnamoyl amide derivatives exhibit in vitro inhibitory activity against NagZ and show synergistic antibacterial effects when combined with β-lactam antibiotics [14], the repertoire of available NagZ inhibitors remains limited, and their application in foodborne pathogens has not yet been explored.

In a previous study, Liu et al. [15] have demonstrated that the regulatory function of NagZ exhibits pronounced genetic background dependence. However, whether NagZ contributes to environmental stress tolerance, and whether exogenous substrate GlcNAc can modulate antibiotic susceptibility, has not been investigated. To address these questions, this study systematically evaluated the impact of NagZ on antibiotic resistance in *Y. enterocolitica* and explored the synergistic effect of combining GlcNAc with β-lactam antibiotics and fosfomycin sodium. Furthermore, by constructing a *nagZ* deletion mutant, the role of this gene in environmental stress tolerance was examined. This study aims to elucidate the dual function of NagZ in environmental adaptation and resistance regulation in *Y. enterocolitica*, as well as the modulatory effect of exogenous GlcNAc intervention on antibiotic susceptibility, providing a theoretical basis for the development of novel NagZ-targeted antibacterial strategies.

## 2. Materials and Methods

### 2.1. Bacterial Strains, Plasmids, and Culture Conditions

All reagents were purchased from Beijing Solarbio Science & Technology Co., Ltd. (Beijing, China). The bacterial strains and plasmids used in this study are listed in Table 1. *Escherichia coli* DH5α, used as the host bacteria in plasmid construction, was cultured at 37 °C in LB medium (5 g/L yeast extract, 10 g/L tryptone, and 10 g/L NaCl). *Y. enterocolitica* 8081 (biotype 1B and serotype O:8) was used as the parent strain for constructing mutants. *Y. enterocolitica* strains were grown in LB medium without NaCl (designated LBNS) at 26 °C. Chloramphenicol (34 μg/mL) was added as required for relevant mutants.

### 2.2. Plasmid and Strain Construction

The primers used in this study are listed in Table 2. Strain construction was performed following a previously reported method with some modifications [16]. Briefly, the upstream and downstream flanking fragments of the *nagZ* gene were amplified from *Y. enterocolitica* 8081 genomic DNA using the primers listed in Table 2. These fragments were cloned into pDS132 and introduced into *Y. enterocolitica* 8081 by conjugation with *E. coli* S17-1 λpir. Single-crossover integrants were selected, followed by sucrose counter-selection to obtain double-crossover mutants. The deletion was verified by colony PCR and sequencing. This is an in-frame, nonpolar deletion with no disruption of upstream or downstream genes. The upstream and downstream homology arms were 510 bp and 508 bp in length, respectively, and the deleted region comprised the entire 1026 bp coding sequence of the *nagZ* gene (locus tag: BAH-0005080). The pBAD24 plasmid was double-digested with *XbaI* and *HindIII*, and the purified linearized vector was ligated with the *nagZ* gene fragment via homologous recombination at an appropriate molar ratio. The ligation products were then transformed into competent cells for cloning by heat shock, and positive clones were screened by colony PCR using pBAD24-specific primers. To avoid interference with subsequent MIC assays, the backbone of pBAD24 *nagZ* was amplified by inverse PCR, and the chloramphenicol resistance marker gene was cloned into the plasmid via homologous recombination, thereby replacing the original ampicillin resistance with chloramphenicol resistance. Finally, the resulting expression plasmid was introduced into the corresponding deletion mutant by electroporation, yielding the complemented strain. Gene expression in the complemented strain was induced with 0.1% (*w*/*v*) L-arabinose.

### 2.3. Growth Curve Assay

To evaluate the growth kinetics, the wild-type (WT), Δ*nagZ* mutant, and complemented strain Δ*nagZ-nagZ* were cultured in both LB and LBNS media. Strains preserved at −80 °C were first streaked separately onto LB and LBNS agar plates for reactivation. Single colonies were then inoculated into the corresponding LB or LBNS liquid medium and incubated overnight at 26 °C with shaking. The overnight cultures were subsequently diluted to an initial optical density at 600 nm (OD_600_) of 0.05 in 100 mL of LB or LBNS liquid medium contained in 250 mL flasks. The cultures were grown at 26 °C with shaking at 180 rpm for 24 h. Sterile liquid medium was used as a blank control. Cell density was monitored by measuring the absorbance at 600 nm every 2 h using a UV-visible spectrophotometer (UNICO, Dayton, NJ, USA). Growth curves were then constructed based on the recorded OD_600_ values. All experiments were performed with three independent biological replicates for each group.

### 2.4. Biofilm Assays

Crystal violet staining was used to measure biofilm formation. Bacterial cells in the logarithmic growth phase cultivated in LBNS liquid medium were added to 96-well plates, with each well containing 200 μL of bacterial suspension at an initial OD_600_ of approximately 0.05. Uninoculated LBNS liquid medium was used as a blank control. Biofilm formation was assessed after static incubation for 24 h, 48 h, and 72 h at 26 °C. After removal of the planktonic growth medium, the adherent biofilms were washed twice with 220 µL of phosphate-buffered saline (PBS) and fixed with 220 µL of 100% methanol for 15 min after drying at 80 °C for 5 min. The biofilms were then stained with 200 µL of 0.1% crystal violet solution for 5 min, and the bound dye was subsequently solubilized with 200 µL of 33% acetic acid for 10 min at room temperature. The optical density of each well was measured at 595 nm (OD_595_) using a microplate reader. The final OD_595_ value representing biofilm biomass was obtained by subtracting the blank control readings. Six wells on a single plate were used as technical replicates, and three independent biological experiments were performed for each experimental group.

### 2.5. Minimum Inhibitory Concentration Assays

The minimum inhibitory concentration (MIC) was determined to quantitatively evaluate the susceptibility of planktonic bacterial cells to the antibiotics ampicillin, fosfomycin sodium, and ceftazidime. Overnight bacterial cultures were inoculated at a ratio of 1% (*v*/*v*) into fresh LBNS liquid medium and incubated until the OD_600_ reached approximately 0.4. The culture was then diluted with sterile LBNS to an OD_600_ of 0.2, followed by a further 1000-fold dilution, yielding a final inoculum of approximately 5 × 10^5^ CFU/mL, which was confirmed by viable plate counting. Subsequently, 200 μL of the diluted bacterial suspension was mixed with 200 μL of LBNS containing the corresponding antibiotic at 512 μg/mL. Two-fold serial dilutions were then performed to obtain final antibiotic concentrations ranging from 0, 0.25, 0.50, 1.00, 2.00, 4.00, 8.00, 16.00, 32.00, 64.00, 128.00, to 256.00 μg/mL. Aliquots of 200 μL from each dilution were transferred into 96-well plates. Three independent biological replicates were set up for each experimental group, with five technical replicates per biological replicate. Modal MIC values were reported based on three biological replicates. The plates were incubated at 26 °C for 24 h, after which turbidity in each well was visually inspected. The MIC was defined as the lowest antibiotic concentration that completely inhibited visible bacterial growth. For MIC determination in the presence of exogenous GlcNAc, the assay was performed identically, except that the antibiotic-containing LBNS medium was supplemented with 2% GlcNAc prior to the test. This concentration was selected based on previous studies demonstrating that exogenous GlcNAc can effectively modulate β-lactam susceptibility in Gram-negative bacteria [17].

### 2.6. Environmental Stress Survival Assays

A single colony was inoculated into 5 mL of LBNS liquid medium and cultured overnight at 26 °C with shaking at 180 rpm. The overnight culture was transferred into 100 mL of LBNS liquid medium at an initial OD_600_ of approximately 0.05 and incubated until the mid-logarithmic phase (OD_600_ ≈ 0.4). The resulting bacterial suspension was then divided into equal aliquots and subjected to the following treatments: for temperature stress, aliquots were incubated statically at 4 °C or 42 °C for 1 h, with 26 °C static incubation for 1 h set as the corresponding temperature control; for chemical stress, aliquots were diluted 50-fold with LBNS liquid medium containing 0.2% (*w*/*v*) SDS or 10 mM H_2_O_2_ and incubated at 26 °C for 1 h. Control groups were treated with sterile water under the same dilution and incubation conditions. After treatment, each bacterial suspension was serially diluted 10-fold, ranging from 10^−3^ to 10^−6^. An appropriate dilution was then spread onto LBNS agar plates and incubated at 26 °C for 48 h, after which colony-forming units (CFUs) were counted. All experiments were performed with three independent biological replicates; three agar plates were counted for each dilution within a single replicate. Survival rates (%) were calculated using the following formula: Survival rate (%) = (CFU of treatment group/CFU of control group) × 100%, where the control group consisted of bacterial suspensions treated identically but with sterile water replacing the stressors.

### 2.7. Statistical Analysis

All statistical analyses and graphical presentations were performed using GraphPad Prism (v.8.0.1) software. Data are expressed as the mean ± standard deviation (SD). Statistical comparisons were conducted using one-way ANOVA followed by Tukey’s post hoc test. In all analyses, *p* ≤ 0.05 was considered statistically significant, and exact *p*-values are provided in the text or figure legends where possible. Significance markers (*, *p* < 0.05; **, *p* < 0.01; ***, *p* < 0.001) are annotated on all figures. In all assays, *n* refers to the number of independent biological replicates.

## 3. Results

### 3.1. Effects of the nagZ Gene on Growth and Biofilm Formation in Y. enterocolitica

**Figure 1:**
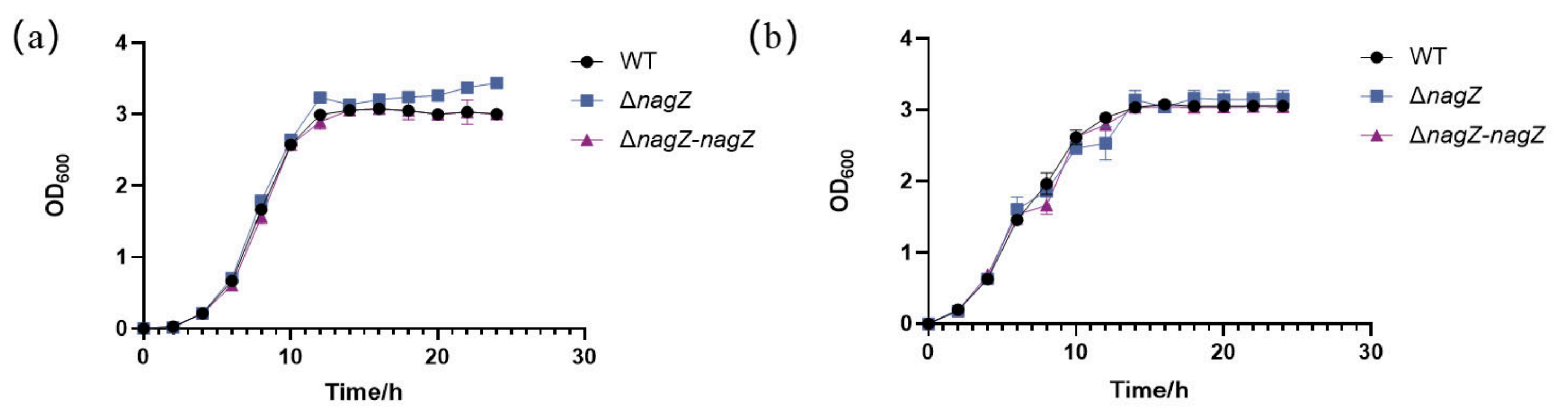
Determination of growth curves of WT, mutant, and complementation strains of *Y. enterocolitica.* (**a**) Growth curve of strains in LB medium. (**b**) Growth curve of strains in LBNS medium. *n* = 3. Data are average values and standard deviations of triplicate experiments.

The growth curves of the WT, *nagZ* deletion mutant (Δ*nagZ*), and complemented strain (Δ*nagZ-nagZ*) were determined in both LB and LBNS media (Figure 1). In LB medium, the deletion mutant exhibited a marginally higher maximum biomass OD_600_ than the WT strain; however, this difference was not statistically significant (*p* > 0.05). The complemented strain displayed a growth profile comparable to that of the WT, suggesting that *nagZ* exerts only a minor effect on the growth of *Y. enterocolitica* under nutrient-rich conditions (Figure 1a). Given that *Y. enterocolitica* exhibits limited tolerance to elevated NaCl concentrations [16] growth curves of the three strains were further assessed in low-salt LBNS medium. No significant differences in growth trends were observed among the strains under this condition (Figure 1b).

**Figure 2:**
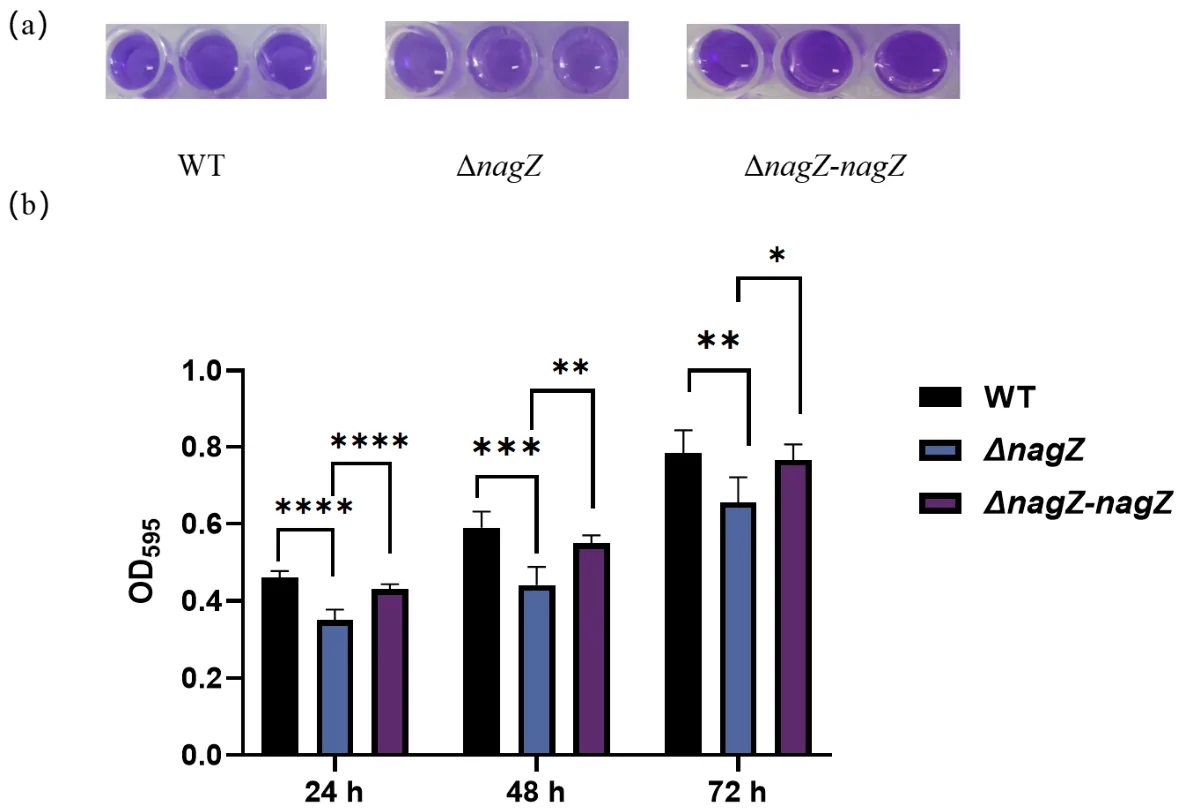
Biofilm formation ability of WT, Mutant, and complementation strains of *Y. enterocolitica*. (**a**) Crystal violet staining images of biofilms formed after 72 h. The plate layout for the staining images is as follows: WT; Δ*nagZ*; Δ*nagZ-nagZ*, from left to right. (**b**) Quantitative analysis of biofilm formation (OD_595_) at 24 h, 48 h, and 72 h. *n* = 3; data are average values and standard deviations of the experiment (*, *p* < 0.05; **, *p* < 0.01; ***, *p* < 0.001; ****, *p* < 0.0001).

In this study, biofilm formation was assessed using crystal violet staining in 96-well plates. Figure 2a displays biofilm biomass at 72 h. Biofilm formation of the Δ*nagZ* mutant was significantly reduced compared with that of the WT strain (*p* < 0.05). The absolute OD_595_ values (mean ± SD) for the Δ*nagZ* mutant were 0.35 ± 0.03, 0.44 ± 0.05, and 0.66 ± 0.07 at 24 h, 48 h, and 72 h, respectively; the corresponding WT values were 0.46 ± 0.02, 0.59 ± 0.04, and 0.79 ± 0.06. These results correspond to decreases of 23.91%, 25.42%, and 16.46% (*p* < 0.0001, *p* < 0.001, and *p* < 0.01, respectively) relative to the WT strain at each time point. Notably, the complemented strain Δ*nagZ-nagZ* restored biofilm formation to levels comparable to those of the WT (Figure 2b). These findings indicate that the *nagZ* gene is involved in the regulation of biofilm formation in *Y. enterocolitica*, and may indirectly influence the bacterial response to external environmental stimuli.

### 3.2. Effects of the nagZ Gene on Antibiotic Resistance in Y. enterocolitica

To investigate the impact of NagZ on antibiotic resistance in *Y. enterocolitica*, the minimum inhibitory concentrations (MICs) of ampicillin, ceftazidime, and fosfomycin sodium against the WT, Δ*nagZ* mutant strains, and complementation strains were determined using the broth microdilution method.

As shown in Table 3, a substantial MIC reduction was observed for ampicillin in the Δ*nagZ* mutant relative to WT, while the two-fold MIC shifts for ceftazidime and fosfomycin sodium fell within the accepted ±one-dilution experimental error inherent to broth microdilution assays. Given that the enzymatic function of NagZ is to hydrolyze GlcNAc-containing peptidoglycan fragments, exogenous GlcNAc was added to observe its impact on MICs. The MICs of strains to three antibiotics in both the WT, Δ*nagZ* strains and Δ*nagZ-nagZ* strains in the presence of exogenously added GlcNAc were measured. The results showed that, in the WT strain, the addition of GlcNAc reduced the MICs to levels comparable to those observed in the Δ*nagZ* mutant. Furthermore, in the Δ*nagZ* background, the addition of GlcNAc led to a further reduction in the MIC of ampicillin only, whereas no additional MIC change was observed for fosfomycin or ceftazidime. In the complemented strain, the addition of exogenous GlcNAc had no different effect from that in the WT strain for the three antibiotics.

### 3.3. Effects of the nagZ Gene on Environmental Tolerance in Y. enterocolitica

Bacterial environmental tolerance is crucial for survival and pathogenicity. In this study, temperature, envelope stress (SDS), and oxidative stress (H_2_O_2_) were selected as evaluation indicators to investigate the effects of *nagZ* deletion and complementation on the environmental adaptability of *Y. enterocolitica*. Temperature is a key environmental determinant influencing the survival, growth, and virulence of *Y. enterocolitica* [18].

**Figure 3:**
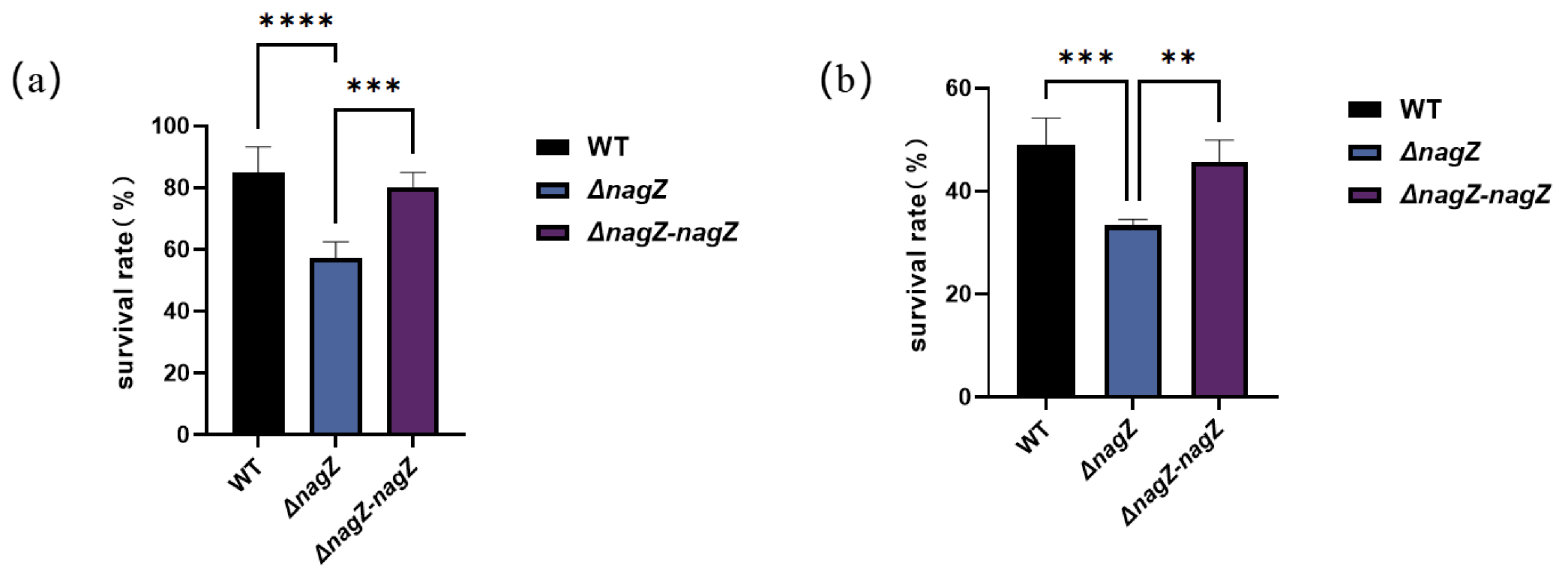
Effects of temperature on the survival rate of different bacterial strains of WT, mutant, and complementation strains of *Y. enterocolitica.* (**a**) Survival rate of strains at 42 °C. (**b**) Survival rate of strains at 4 °C. *n* = 3; data are average values and standard deviations of the experiment (**, *p* < 0.01; ***, *p* < 0.001; ****, *p* < 0.0001).

As shown in Figure 3, all three strains survived at both 42 °C and 4 °C. However, the Δ*nagZ* mutant exhibited markedly lower survival than the WT strain at both temperatures, with survival rates significantly decreasing from 85.12 ± 8.30% to 57.38 ± 5.20% at 42 °C (*p* < 0.0001) and from 49.02 ± 5.30% to 33.38 ± 1.20% at 4 °C (*p* < 0.001). The complemented strain Δ*nagZ-nagZ* nearly restored survival to levels comparable to those of the WT at both temperatures, with survival rates of 80.34 ± 4.60% at 42 °C and 45.65 ± 4.32% at 4 °C. These findings indicate that *nagZ* contributes to tolerance of *Y. enterocolitica* under both high-temperature and cold stress.

**Figure 4:**
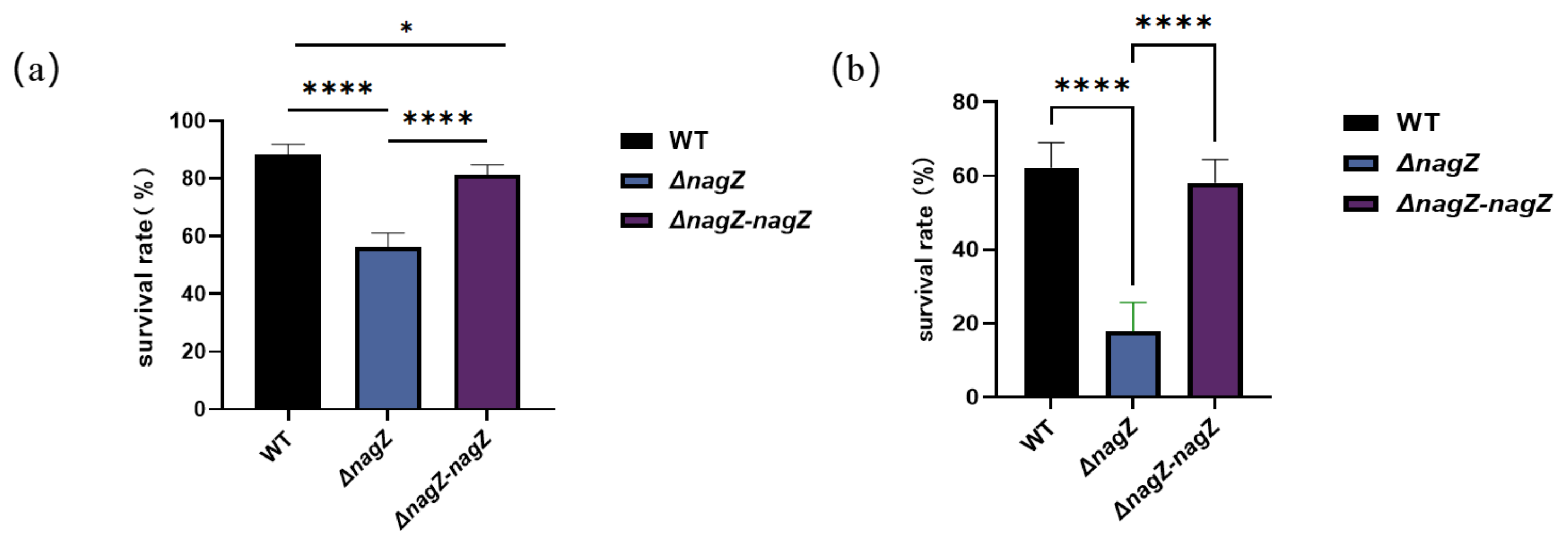
Effects of SDS and oxidative stress on the survival rate of different bacterial strains of WT, mutant, and complementation strains of *Y. enterocolitica.* (**a**) Survival rate of strains under SDS. (**b**) Survival rate of strains under oxidative stress. *n* = 3; data are average values and standard deviations of the experiment (*, *p* < 0.05; ****, *p* < 0.0001).

In this study, 0.2% SDS was added to the culture medium to establish an envelope stress model, allowing systematic evaluation of strain survival and adaptability under such environmental pressure. As shown in Figure 4a, deletion of *nagZ* significantly reduced strain survival under SDS, with the survival rate decreasing from 88.26 ± 3.58% to 56.16 ± 5.05% (*p* < 0.001). The complemented strain partially restored survival, reaching 81.27 ± 3.58%, which was significantly higher than that of the Δ*nagZ* mutant but still lower than that of the WT strain (*p* < 0.05). These results indicated that loss of *nagZ* markedly compromises the tolerance of *Y. enterocolitica* to SDS-induced envelope stress, suggesting that NagZ plays an important role in defending against detergent-induced damage.

Meanwhile, oxidative stress was simulated by supplementing the culture medium with 10 mM H_2_O_2_. As shown in Figure 4b, deletion of *nagZ* significantly reduced strain survival under oxidative stress, with the survival rate decreasing from 62.16 ± 6.86% to 17.86 ± 7.77% (*p* < 0.0001). The complemented strain restored survival to a level comparable to that of the WT strain, with a survival rate of 58.12 ± 6.30% (*p* > 0.05). This suggests that *nagZ* is involved in the bacterial response to oxidative stress, and its loss impairs the capacity of the strain to scavenge ROS or repair oxidative damage, thereby reducing survival under such conditions.

## 4. Discussion

The ongoing dissemination of antibiotic resistance, coupled with enhanced environmental stress tolerance, poses a dual challenge to the prevention and control of foodborne pathogens. A surveillance study of retail meat and meat products in South Africa reported resistance frequencies of 94% for ampicillin, 83% for cephalothin, and 41% for amoxicillin. The high resistance to ampicillin and cephalothin is largely intrinsic in *Y. enterocolitica*, which may explain these frequencies [19]. Concurrently, its environmental tolerance is equally notable: biofilm formation rates among isolates have been reported as high as 77.8%, with strong biofilm-forming strains showing a gradient-dependent increasing trend along the food chain [20]. Therefore, identifying key molecular determinants that modulate antibiotic susceptibility and environmental adaptability in *Y. enterocolitica* is of critical importance for designing effective intervention strategies. NagZ, a core hydrolase in the peptidoglycan recycling pathway, has been demonstrated to participate in the regulation of β-lactamase expression and biofilm homeostasis in various Gram-negative NagZ-dependent bacteria [21]. However, recent studies across different bacterial species have revealed that NagZ function exhibits marked genetic background dependence, with some reports even showing opposing regulatory modes. In *Stenotrophomonas maltophilia*, both NagZ-dependent and NagZ-independent activating ligands have been identified, and different β-lactam antibiotics exhibit distinct preferences for these two signaling pathways [22]. Studies in *E. cloacae* have further shown, from a clinical perspective, that sub-inhibitory concentrations of cefazolin and imipenem induce AmpC expression and enhance resistance in a NagZ-dependent manner, and that *nagZ* deletion completely abolishes this effect [23]. Nevertheless, the functional role of this gene in *Y. enterocolitica* has not been fully characterized. Given the unique ecological niche and temperature-dependent virulence characteristics of *Y. enterocolitica*, its cell wall metabolism regulatory network may differ substantially from those of other enteric bacteria. In this study, we systematically investigated the responses of *Y. enterocolitica* to antibiotic and environmental stresses by constructing a gene deletion mutant and a complemented strain, thereby extending current knowledge regarding the roles of *nagZ* under environmental stress and exogenous GlcNAc conditions within the genus *Yersinia*.

In this study, although the maximum OD_600_ of the Δ*nagZ* mutant in LB medium was higher than that of the WT strain, the difference was not statistically significant. Similarly, no significant growth difference was detected in LBNS medium. These results indicate that deletion of *nagZ* does not significantly affect the basal growth of *Y. enterocolitica*. Consequently, the contribution of this growth-related phenotype to the overall environmental tolerance assessment is relatively limited. In contrast, *nagZ* deletion attenuated the biofilm-forming capacity of the strain. Biofilm formation is a critical factor enabling foodborne pathogens to colonize food-processing surfaces and establish persistent contamination. The reduced biofilm-forming ability observed in the mutant implies that the strain may be less efficient in establishing contamination reservoirs via surface attachment during slaughtering, transportation, and storage, thereby potentially facilitating the efficacy of cleaning and disinfection measures. Notably, the structural integrity of biofilms is closely associated with bacterial tolerance to antibiotics: the extracellular polymeric substance (EPS) matrix not only physically impedes antibiotic penetration but also creates a microenvironment conducive to horizontal gene transfer of resistance determinants. Based on this rationale, the role of *nagZ* in modulating antibiotic susceptibility was further investigated.

For antibiotic susceptibility testing, three antibiotics, ampicillin, ceftazidime, and fosfomycin sodium, were selected. The Δ *nagZ* mutant displayed a marked reduction in ampicillin MIC compared with the WT strain, whereas the two-fold MIC alterations for ceftazidime and fosfomycin sodium were within the ±one-dilution experimental error typical of broth microdilution assays. Among the three tested agents, only ampicillin showed a robust phenotypic difference upon *nagZ* deletion, suggesting that *nagZ* positively mediates ampicillin resistance in *Y. enterocolitica*. This ampicillin-specific phenotype is consistent with the finding that NagZ promotes AmpC β-lactamase-mediated resistance in *E. cloacae* [24]. The *Y. enterocolitica* 8081 strain used in this study stably harbors these two canonical chromosomal resistance determinants, together with three putative metallo-β-lactamase homologs (YE1559, YE2635, and YE0055) [8,25]. This intrinsic β-lactamase background provides the core molecular basis for NagZ-mediated resistance modulation in *Y. enterocolitica*. It is proposed that the reduced β-lactam resistance in the Δ*nagZ* mutant arises from diminished production of NagZ-dependent peptidoglycan-derived signaling ligands. Depletion of these molecules attenuates AmpR-mediated transcriptional activation of downstream β-lactamase genes, consequently reducing the overall β-lactam hydrolytic capacity and increasing antibiotic susceptibility. Studies in *P. aeruginosa* and *E. cloacae* have demonstrated that NagZ catalyzes the generation of 1,6-anhydromuropeptides, which act as signaling ligands to activate the transcriptional regulator AmpR, thereby driving *ampC* expression and conferring β-lactam tolerance [21,26]. Although the MIC changes for ceftazidime and fosfomycin sodium were within the accepted experimental error, a consistent numerical decrease was observed for both agents. This trend may suggest that *nagZ* deletion has a broader but weaker effect on antibiotic susceptibility than that observed for ampicillin. For ceftazidime, the same NagZ–AmpR–AmpC signaling axis proposed for ampicillin could partially contribute to the observed trend, since ceftazidime is also a β-lactam substrate of AmpC. For fosfomycin sodium, the slight decrease may be attributable to metabolic perturbation triggered by impaired peptidoglycan recycling, in which loss of NagZ alters intracellular amino sugar phosphate homeostasis and may competitively modulate the transport efficiency of GlpT/UhpT, thereby changing intracellular fosfomycin accumulation [27,28,29,30]. These interpretations remain speculative, and further experimental validation is required to confirm whether the observed trends are mechanistically meaningful. These mechanisms remain speculative and require further experimental validation. From a food safety standpoint, the restored ampicillin susceptibility conferred by *nagZ* deletion suggests that intervention strategies targeting NagZ might contribute to reducing selective pressure and warrant further investigation as a strategy to limit the dissemination of resistant strains along the food chain.

Currently, NagZ has emerged as a highly promising broad-spectrum antimicrobial target, and its specific inhibitors are expected to be developed as novel adjuvant therapeutic strategies to restore or enhance the clinical efficacy of β-lactam antibiotics. Several NagZ-targeting approaches have been successively reported: PUGNAc blocks NagZ function at the enzymatic level, and its highly selective derivative N-pentanoyl-PUGNAc further improves selectivity for NagZ over functionally related human enzymes [31,32]; the cinnamamide derivatives 11h and Br-6, when combined with imipenem or ceftazidime, exhibit significant synergistic effects and effectively restore drug susceptibility in *P. aeruginosa* [14]; and peptide-conjugated peptide nucleic acids (PPNAs) targeting *nagZ* mRNA achieve intervention at the gene expression level, reducing the ceftazidime MIC from 64 μg/mL to 8 μg/mL [33]. Collectively, these studies validate the feasibility of NagZ as an antimicrobial target. Notably, exogenous supplementation with GlcNAc, a natural substrate analogue of NagZ, reduced the MIC of the WT strain to a level comparable to that of the *nagZ* deletion mutant. Furthermore, when GlcNAc was added to the deletion mutant background, an even greater reduction in MIC was observed, suggesting a further effect between exogenous GlcNAc intervention and genetic disruption of *nagZ*. We cannot rule out NagZ-independent confounders such as osmotic pressure, global metabolic shifts, or subtle growth alterations caused by high-dose GlcNAc. Follow-up work incorporating growth kinetic controls across a range of GlcNAc concentrations, alongside formal interaction statistical analysis, will be required to dissect whether GlcNAc modulates peptidoglycan-recycling-associated signaling pathways. From a food safety and control perspective, GlcNAc is a naturally occurring amino sugar with a favorable safety profile. This finding at 2% GlcNAc provides mechanistic insight under laboratory conditions; its practical feasibility as an exogenous additive or processing aid remains to be validated in food matrices and at concentrations relevant to real food-processing scenarios. Interpretation of the 2% exogenous GlcNAc supplementation experiments is constrained in this work.

Beyond its role in antibiotic resistance modulation, this study further revealed that NagZ is involved in the response to multiple environmental stresses. Deletion of *nagZ* significantly compromised bacterial tolerance to extreme temperatures, surfactant exposure, and oxidative stress, suggesting that NagZ plays an indispensable role in the bacterial defense against diverse environmental challenges. For example, although *Y. enterocolitica* can grow slowly at refrigeration temperatures following long-term acclimation, an abrupt temperature downshift from 26 °C to 4 °C may trigger transient membrane rigidification and translational arrest, leading to a temporary loss of culturability [34]. The Δ*nagZ* mutant may be more susceptible to such temperature shifts, consistent with its significantly reduced tolerance to extreme temperatures. Mechanistically, as a core hydrolase in the peptidoglycan recycling pathway, loss of NagZ function may lead to aberrant accumulation of cell wall metabolites or depletion of signaling molecules, thereby impairing adaptive responses under stress conditions. Notably, the impact of NagZ on environmental tolerance may involve regulatory pathways independent of the AmpC β-lactamase.

Despite these findings, this study has several limitations. First, experiments were performed using a single *Y. enterocolitica* 8081 strain background; phenotypic responses may vary across different isolates. Second, AmpC expression and enzyme activity were not directly measured, so the mechanistic link between NagZ and AmpC-mediated β-lactam resistance remains inferred from phenotypic results. Third, for the complemented strain, *nagZ* expression in Δ*nagZ-nagZ* was not verified by RT-qPCR or NagZ enzymatic activity, and no empty-vector control was included in any assay. Without this control, the potential phenotypic contributions from the pBAD24 plasmid backbone and chloramphenicol resistance marker cannot be excluded. Fourth, MIC determinations were conducted in LBNS medium at 26 °C for 24 h. This condition was selected to mimic typical food-associated environmental temperatures and represents the optimal growth temperature for *Y. enterocolitica*. Nevertheless, this setup deviates from the conditions recommended by the Clinical and Laboratory Standards Institute (CLSI) and the European Committee on Antimicrobial Susceptibility Testing (EUCAST), namely, cation-adjusted Mueller-Hinton broth at 35 °C for 16–20 h. Accordingly, the obtained MIC values cannot be directly compared with clinical breakpoints defined by CLSI or EUCAST. This study characterizes the dual regulatory roles of the *nagZ* gene in *Y. enterocolitica* 8081 in environmental adaptation and antibiotic susceptibility, explores the modulatory effect of GlcNAc on antibiotic sensitivity, and, for the first time, reveals its impact on biofilm formation. Future investigations will include quantitative detection of AmpC expression, validation of *nagZ* expression and NagZ enzymatic activity in complemented strains, inclusion of empty-vector controls, testing with an expanded strain panel, standardized susceptibility assays complying with CLSI/EUCAST guidelines, and food-matrix-based validation to further verify the current findings.

## 5. Conclusions

Overall, this study provides new insights into NagZ function in *Y. enterocolitica* 8081. The deletion of *nagZ* compromised biofilm formation and survival under multiple stresses, including heat, cold, SDS, and oxidative stress, and exogenous GlcNAc reduced ampicillin MICs, with a further reduction observed in the Δ*nagZ* background. These findings suggest that, within this strain background, NagZ may act as a dual regulator linking β-lactam resistance and environmental adaptability. Our work also lays the groundwork for future investigations into GlcNAc-based metabolic interventions and dual-target strategies aimed at both antimicrobial resistance and environmental adaptability.

## Figures and Tables

**Table 1:** Bacterial strains and plasmids used in this study.

Strains and Plasmids	Relevant Characteristics	Sources
*Y. enterocolitica*		
WT	WT, Serotype O:8, Biotype 1B (strain 8081), pYV	Lab stocked
Δ*nagZ*	WT; Δ*nagZ*	This study
Δ*nagZ-nagZ*	WT; Δ*nagZ*, pBAD24-*nagZ*; Cm^r^	This study
*E. coli*		
S17-1 λpir	*RP4-2*(*Km::Tn7*, *Tc::Mu-1*), *pro-82*, *LAMpir*, *recA1*, *endA1*, *thiE1*, *hsdR17*, *creC510*	Lab stocked
DH5α	F^−^, φ80, *lacZ*ΔM15, Δ(*lacZYA-argF*) U169, *deoR*, *recA1*, *endA1*, *hsdR17*(*rk^−^*, *mk^+^*), *phoA*, *supE44*, *λ^−^*, *thi-1*, *gyrA96*, *relA1*	Lab stocked
Plasmids		Lab stocked
pDS132	conditional replication vector; R6K origin, mobRK4 transfer origin, sucrose-inducible-*sacB*; Cm^r^	Lab stocked
pDS132-*nagZ*	upstream and downstream fragments of *nagZ* gene (locus tag: BAH-0005080) were cloned into pDS132; Cm^r^	This study
pBAD24	*AraC*, promoter PBAD; Amp^r^	Lab stocked
pBAD24-*nagZ*	*AraC*, PBAD*nagZ*; Cm^r^	This study

Amp, ampicillin; Cm, chloramphenicol; ^r^, resistance.

**Table 2:** Primers used in this study.

Primers	Sequence (5′–3′)	Relevant Experiment
*nagZ*-UF	ACTGGCACCCCTGCATATGGATAT	Cloning of *nagZ* gene upstream
*nagZ*-UR	GTAGGTAGACTATCATATGACAACCTCACATAATTCCC	Cloning of *nagZ* gene upstream
*nagZ*-DF	AAGGGGAATTATGTGAGGTTGTCATATGATAGTCTACCTAC	Cloning of *nagZ* gene downstream
*nagZ*-DR	TGAGGTGAAAGATCTTTAAA	Cloning of *nagZ* gene downstream
24-F	ATGCCATAGCATTTTTATCC	Validation of the *nagZ* gene
24-R	GATTTAATCTGTATCAGG	Validation of the *nagZ* gene
24bone-F	CAGTGATTTTTTTCTCCATACTCTTCCTTTTTCAATATTAT	Cloning of pBAD24 bone
24bone-R	TGGCAGGGCGGGGCGTAACTGTCAGACCAAGTT	Cloning of pBAD24 bone
Cm-F	TTGAAAAAGGAAGAGTATGGAGAAAAAAATCACTGG	Cloning of chloramphenicol marker gene
Cm-R	TGGCAGGGCGGGGCGTAACTGTCAGACC	Cloning of chloramphenicol marker gene
*nagZ*-F	TCACCATGGTACCCGGGGATCCTCTAGAGTGGGTCCAGTAATGTTAGAC	Cloning of *nagZ* gene
*nagZ*-R	ATCTTCTCTCATCCGCCAAAACAGCCAAGCTTTTATCCTCGCGACTTATGTGCATC	Cloning of *nagZ* gene

**Table 3:** Antibiotic resistance of *Y. enterocolitica* strains. (*n* = 3).

Strain/Treatment	MIC/(μg/mL)		
	Ampicillin	Fosfomycin Sodium	Ceftazidime
WT	64	128	128
Δ*nagZ*	16	64	64
Δ*nagZ-nagZ*	64	128	128
WT + GlcNAc	16	64	64
Δ*nagZ* + GlcNAc	2	64	64
Δ*nagZ-nagZ* + GlcNAc	16	64	64

## Data Availability

The raw data supporting the conclusions of this article will be made available by the authors upon reasonable request.

## Abbreviations

The following abbreviations are used in this manuscript:

*Y. enterocolitica*	*Yersinia enterocolitica*
GlcNAc	N-acetylglucosamine
*P. aeruginosa*	*Pseudomonas aeruginosa*
*E. cloacae*	*Enterobacter cloacae*
*E. coli*	*Escherichia coli*
SDS	Sodium dodecyl sulfate
MIC	Minimum inhibitory concentration
NagZ	β-N-acetylglucosaminidase
AmpC	AmpC β-lactamase
WT	Wild type
PBS	Phosphate-buffered saline
CFU	Colony-forming unit
OD	Optical density
ROS	Reactive oxygen species
EPS	Extracellular polymeric substances
PNA	Peptide nucleic acid
PPNA	Peptide-conjugated peptide nucleic acid
SD	Standard deviation
GlpT	Glycerol-3-phosphate transporter
UhpT	Hexose-6-phosphate transporter
BlaA	Class A β-lactamase
BlaB	Class C β-lactamase
CLSI	Clinical and Laboratory Standards Institute
EUCAST	European Committee on Antimicrobial Susceptibility Testing
